# Biomechanical insights into gait rehabilitation for multiple sclerosis: a narrative review of exercise modalities and progressive training approaches

**DOI:** 10.1186/s13102-025-01339-4

**Published:** 2025-10-14

**Authors:** Sara Sepehrifar, Mansour Sahebozamani

**Affiliations:** Department of Sports Injuries and Corrective Exercises, Faculty of Sports Science, University of Shahid Bahonar Kerman, Kerman, Iran

**Keywords:** Multiple sclerosis, Gait rehabilitation, Exercise, Biomechanics, Progressive training

## Abstract

**Introduction:**

Gait dysfunction is a pervasive and debilitating symptom in people with multiple sclerosis (pwMS), characterized by reduced walking speed, shorter stride length, increased gait variability, and compromised postural control, significantly reducing quality of life. The aim of this study is to examine the effects of various exercise modalities on gait biomechanics, evaluate the role of progressive training principles in optimizing outcomes, and provide an evidence-based framework for individualized gait rehabilitation in MS, with the goal of developing targeted exercise strategies to effectively address the multifactorial nature of gait impairments.

**Objective:**

This narrative review critically examines the biomechanical outcomes of structured exercise interventions for gait rehabilitation in pwMS, focusing on kinematic (e.g., joint angles), kinetic (e.g., ground reaction forces), and spatiotemporal (e.g., stride length, gait speed) parameters. It also assesses the role of progressive training principles, such as overload and task specificity, in optimizing functional outcomes.

**Methods:**

A comprehensive literature search was conducted in PubMed, Web of Science, and Scopus (2005 to February 2025) following PRISMA guidelines. Randomized controlled trials (RCTs) and non-randomized trials evaluating structured exercise interventions targeting gait biomechanics in pwMS were included. Although this is a narrative review, a PRISMA-style flowchart was used to transparently illustrate the literature selection process.

**Results:**

The review included 15 RCTs and 6 non RCTs with sample sizes ranging from 8 to 50 participants, covering resistance training, aerobic conditioning, balance exercises, cognitive-motor training, and multimodal approaches. Key findings indicated that resistance training improved joint torque and stride length, aerobic training enhanced gait speed and endurance, and multimodal protocols yielded the most comprehensive biomechanical benefits.

**Conclusion:**

High-intensity, task-specific training significantly improves gait biomechanics in pwMS, but the heterogeneity in study designs, small sample sizes, and limited follow-up periods highlight the need for more standardized RCTs. Personalized, progressive training remains essential for optimizing gait rehabilitation outcomes in this population.

**Supplementary Information:**

The online version contains supplementary material available at 10.1186/s13102-025-01339-4.

## Background

Multiple sclerosis (MS) is a chronic inflammatory neurodegenerative disease that leads to the demyelination of nerve fibers in the brain and spinal cord. This damage interferes with the transmission of nerve signals, resulting in unpredictable motor, sensory, and cognitive impairments. Common symptoms include muscle weakness, balance and coordination difficulties, numbness, and memory or attention problems. MS is also often associated with physical and psychological comorbidities, such as fatigue, pain, depression, and anxiety, which can further impact daily functioning and quality of life [1, 2]. These pathological processes lead to significant impairment in neural conduction, resulting in a wide array of motor, cognitive and sensory deficits. Due to the combined impact of reduced physical and cognitive function, as many as 75% of individuals with MS face challenges with balance and walking, which can occur both in the early and later stages of the disease [3]. In individuals with MS, gait is typically marked by increased variability in lower limb movements, reduced stride length, slower walking speed, and an extended duration of double limb support when compared to healthy individuals [4].

In the past, individuals with MS were often discouraged from engaging in exercise out of concern that it might worsen their condition. However, growing evidence has highlighted the potential benefits of physical activity in managing symptoms and enhancing quality of life, which has led to the development of exercise guidelines specifically for people with MS [5]. Regular exercise has been shown to improve muscle strength, balance, coordination, reduce fatigue, and positively influence neuroplasticity, thereby enhancing overall functional capacity and quality of life [6, 7].

Recent studies have focused on the biomechanical underpinnings of these interventions, analyzing changes in kinematic (e.g., joint angles and movement trajectories), kinetic (e.g., ground reaction forces, joint moments), and spatiotemporal (e.g., cadence, stride length, stance time) gait parameters [2, 3, 8, 9]. These parameters provide objective, quantifiable markers for evaluating intervention efficacy, offering a more precise understanding of how specific exercise strategies influence gait mechanics. For instance, strength training enhances lower limb muscle force and joint stabilization, aerobic exercise supports endurance and fatigue resistance, and Sensory motor training improves postural control and proprioceptive feedback [10, 11]. Additionally, cognitive-motor training, such as dual-task and virtual reality-based approaches, has emerged as a promising method for integrating executive function with motor planning, which is particularly relevant for real-world ambulation [10].

Despite this progress, significant gaps remain in the literature. Many previous reviews have broadly confirmed the benefits of exercise for gait function in pwMS, but few have systematically examined the discrete biomechanical changes induced by different exercise modalities or addressed the critical role of progressive training principles, such as overload progression, intensity modulation, and specificity, in promoting lasting neuromuscular adaptations [12].

This narrative review seeks to address these gaps by synthesizing current evidence on the biomechanical effects of diverse exercise protocols in pwMS. Specifically, it aims to: (1) examine the impact of various training modalities on kinematic, kinetic, and spatiotemporal gait parameters (2), evaluate the role of progressive training principles in optimizing biomechanical outcomes, and (3) provide a comprehensive, evidence-based framework for individualized gait rehabilitation in MS. By integrating insights from recent studies, this review aims to support the development of targeted, biomechanically informed exercise strategies that can more effectively address the multifactorial nature of gait impairment in pwMS.

**Fig. 1:**
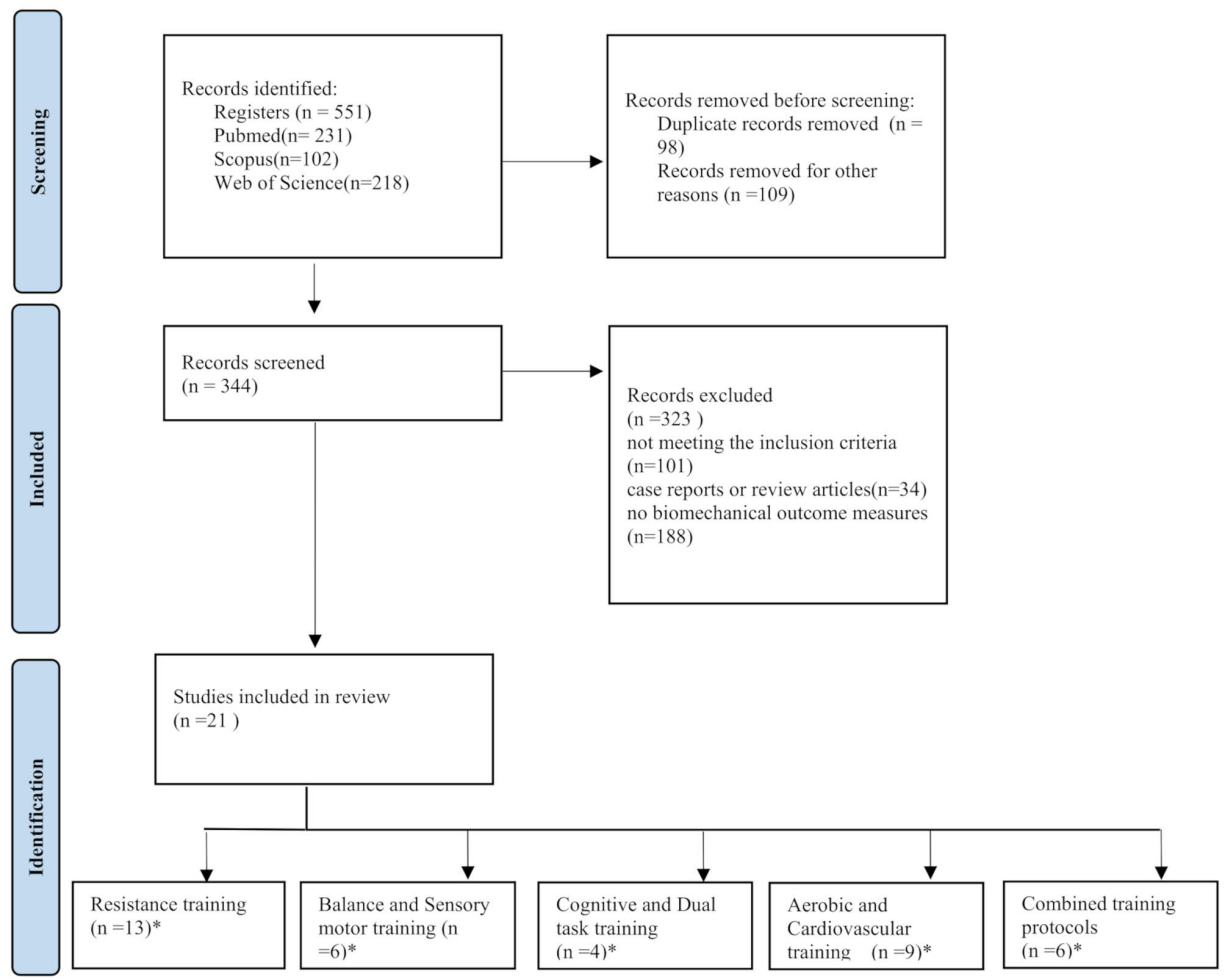
Flowchart of the study screening process *There is some overlap among the reviewed articles in the assigned categories

## Methods

### Study design

The aim of this study is to examine the effects of various exercise modalities on gait biomechanics, evaluate the role of progressive training principles in optimizing outcomes, and provide an evidence-based framework for individualized gait rehabilitation in MS, with the goal of developing targeted exercise strategies to effectively address the multifactorial nature of gait impairments.

### Quality assessment

To evaluate the methodological quality of the included studies, two standardized appraisal tools were employed. Randomized controlled trials (RCTs) were assessed using the Critical Appraisal Skills Programme (CASP) checklist, which consists of 11 questions addressing the research question, randomization, blinding, and validity of results. Scores ranged from 0 to 11, with blinding considered a key criterion. Non-RCTs were evaluated using the Methodological Index for Non-Randomized Studies (MINORS), which includes 12 items rated from 0 to 2 (total score: 0–24). This tool examines methodological quality, risk of bias, and reporting of outcomes. Studies with CASP scores ≥ 8 or MINORS scores ≥ 16 were considered high quality.

### Literature search strategy

A comprehensive literature search was conducted in PubMed, Web of Science, and Scopus for studies published between 2005 and February 2025, following the Preferred Reporting Items for Systematic Reviews and Meta-Analyses (PRISMA) guidelines. We limited our search to this timeframe because more recent studies are more likely to employ standardized methodologies and outcome measures, thereby improving comparability across trials. Although this study is a narrative review, the PRISMA (Preferred Reporting Items for Systematic Reviews and Meta-Analyses) guidelines were followed to enhance the transparency and reproducibility of the literature search and selection process. A PRISMA flow diagram was included to provide a clear visual summary of how studies were identified, screened, and included.

Among 551 total results, 21 studies were included and reviewed. Fig. 1 illustrates the flowchart of the study screening process. The included studies were categorized as follows: resistance training [1, 2, 8, 13–18], Balance and Sensory motor training [2, 15, 19–23]; Aerobic and Cardiovascular training [4, 8, 16–18, 20, 22, 24–26], Cognitive and Dual task training [19, 21, 24, 27] and Combined training protocols [2, 13, 17, 19, 20, 22].

The search strategy was developed using the PICOS framework, focusing on the following elements:

- Population: Adults diagnosed with multiple sclerosis (MS).
- Intervention: Structured exercise programs specifically targeting gait or walking ability.
- Comparator: Usual care, conventional rehabilitation, or alternative exercise interventions.
- Outcomes: Gait-related parameters, including spatiotemporal (e.g., stance duration, stride length, cadence), kinematic (e.g., joint angles, ROM), and kinetic (e.g., muscle forces, ground reaction forces) measures.
- Study Design: Randomized controlled trials (RCTs) and non-Randomized trials published in peer-reviewed journals.

Keywords included combinations of “multiple sclerosis,” “exercise,” “gait,” “walking,” “rehabilitation,” “biomechanics,” and related terms, connected by Boolean operators to capture a broad range of relevant studies. No language or publication date restrictions were applied, but only English-language studies were included to ensure consistency in data interpretation. The complete search strategy, including specific terms and Boolean operators, is provided in Table 1.

**Table 1:** Comprehensive search strategy (PubMed, web of science, Scopus)

Database	Search Terms	Boolean Operators	Date Range	Language	Filters
PubMed	(“Multiple Sclerosis”) AND (“Exercise” OR “Training”) AND (“Gait” OR “Walking” OR “Locomotion”) AND (“Biomechanics” OR “Biomechanical”)	AND, OR	2005 to Feb 2025	English	Peer-reviewed, Full-text
Web of Science	(“Multiple Sclerosis”) AND (“Exercise” OR “Training”) AND (“Gait” OR “Walking” OR “Locomotion”) AND (“Biomechanics” OR “Biomechanical”)	AND, OR	2005 to Feb 2025	English	Peer-reviewed, Full-text
Scopus	(“Multiple Sclerosis”) AND (“Exercise” OR “Training”) AND (“Gait” OR “Walking” OR “Locomotion”) AND (“Biomechanics” OR “Biomechanical”)	AND, OR	2005 to Feb 2025	English	Peer-reviewed, Full-text

### Inclusion and exclusion criteria

- Studies were included if they met the following criteria: 1- Adult participants (≥ 18 years) diagnosed with multiple sclerosis. 2- Implementation of structured exercise programs specifically targeting gait or walking ability, alongside progressive training principles: This includes studies that explicitly incorporate overload progression, intensity modulation, and specificity within their exercise protocols.

3- Use of a control group receiving usual care, conventional rehabilitation, or alternative exercise interventions. 4- Reporting at least one quantitative gait-related outcome (e.g., gait speed, stride length, joint angles). 5- Published as full-text articles in peer-reviewed journals. 6- Studies that include training protocols for a specific duration with over loading progression.

Studies were excluded if they: 1- Used quasi-experimental designs. 2- Included animal models or non-human subjects. 3- Were published as conference abstracts, protocols, or pilot studies without full data. 4- Were not available in English.

### Study selection

The study selection process followed PRISMA guidelines. Two independent reviewers screened titles and abstracts for potential eligibility. Full-text articles were then reviewed for final inclusion, with any discrepancies resolved through consensus or consultation with a third reviewer. Zotero software was used to manage references and remove duplicates, ensuring comprehensive and accurate citation management.

### Data extraction and synthesis

Data from included studies were extracted using a standardized template, capturing key study characteristics, including study design, publication year, sample size, participant demographics (e.g., age, sex, MS subtype, disability level), intervention details (e.g., type, duration, frequency, intensity), and gait-related outcome measures. Key findings, including reported effect sizes and statistical significance, were also documented.

Given the narrative nature of this review, a qualitative synthesis approach was used to identify major themes, methodological strengths and weaknesses, and areas of inconsistency across the included studies. This approach allowed for a more interpretive analysis, capturing both the mechanistic insights and clinical implications of the included studies Table 2.

### Outcome measures

Primary outcomes included quantitative gait parameters, typically assessed using three-dimensional gait analysis systems. These measures were categorized into:

- Spatiotemporal Outcomes: Gait speed, cadence, stride length, step width, stance duration.
- Kinematic Outcomes: Joint angles, range of motion.
- Kinetic Outcomes: Muscle forces, joint torques, ground reaction forces.

Secondary outcomes included functional walking assessments, such as the 10-Meter Walk Test (10MWT) and the 6-Minute Walk Test (6MWT), as well as patient-reported disability scales like the Expanded Disability Status Scale (EDSS) and the Patient-Determined Disease Steps (PDDS) Table 2.

### Ethical considerations

This review relied solely on publicly available, previously published data, eliminating the need for formal ethical approval. However, the ethical implications of gait training interventions in vulnerable populations, such as pwMS, are acknowledged.

## Results

### Study characteristics

A total of 15 randomized controlled trials (RCTs) and 6 non- RCTswere included in this review, with sample sizes ranging from 8 to 50 participants per study. Most studies focused on adults with relapsing-remitting multiple sclerosis (RRMS), though several included individuals with secondary progressive (SPMS) and primary progressive (PPMS) forms of the disease. Intervention durations varied from 4 to 24 weeks, with training frequencies typically 2 to 3 sessions per week. The included studies employed a wide range of exercise protocols, including resistance training, aerobic conditioning, balance exercises, dual-task training, and combined multimodal interventions.

### Quality of included studies

Of the 21 included studies, 15 were RCTs and 6 were non-RCTs. The RCTs scored between 8 and 10 out of 11 on the CASP checklist, indicating robust study designs. Eleven of the 16 RCTs employed single blinding, one lacked blinding, and four had unclear blinding status. Non-RCTs scored between 10 and 11 out of 16 on the MINORS index, reflecting moderate quality. Blinding was challenging in all non-RCTs due to the nature of the interventions. Detailed quality assessments are provided in Tables 6 we1 and 2. Key limitations included the absence of double blinding in RCTs, small sample sizes in some studies, and the lack of a control group in all non-RCTs.

### Resistance training

Resistance training was one of the most extensively studied approaches, primarily targeting lower limb muscle strength, joint stabilization, and postural control. For example, following an 8-week resistance training program, Gutierrez et al. (2005) observed notable pre-to-post intervention improvements in gait parameters among pwMS. In the more-affected limb, participants showed more balanced limb timing, increased stride length (*p* = 0.017), and improved neuromuscular coordination, contributing to greater gait efficiency, reduced risk of falls, and enhanced functional mobility( [13].

Similarly, Filipi et al. (2010) demonstrated that resistance training performed twice weekly over a 6-month period increased joint torque and ground reaction forces, leading to better control and stability during walking. This included increases in peak vertical loading (*p* = 0.018), peak vertical unloading (*p* = 0.017), and vertical loading rate, reflecting a more efficient transfer of body weight during the stance phase. The enhanced braking and propulsive forces observed in this study suggest a more dynamic and controlled gait pattern [14].

In the study by Alashram et al. (2024), pwMS in the 8-week combined Telko and CPT (conventional physical therapy) experimental group showed significant improvements in gait speed and mobility (*P* < 0.05) following the intervention compared to baseline. However, between-group analysis revealed no statistically significant differences between the experimental and CPT-only groups [8]. In the study by Heine et al.(2019), a 16-week program (8 weeks resistance + 8 weeks treadmill endurance) significantly improved walking speed and distance in people with MS compared to the control group [18]. In a study by Manca et al. (2020), individuals with mild to moderate multiple sclerosis (PwMS, EDSS 2–5.5) exhibited a reduced baseline walking speed of 0.8 m/s compared to normative data from healthy subjects (1.4 m/s). Following a 6-week intervention, the direct strength training (DST) group showed a significant increase in walking speed by 0.12 m/s, whereas the contralateral strength training (CST) group demonstrated no significant improvement [28].

However, not all resistance training protocols produced equally robust outcomes. For instance, Callesen et al. (2020) found that 10 weeks PRT improved muscle strength but did not significantly enhance short-distance walking performance or self-perceived walking ability (MSWS-12) compared to balance-based training [15]. Also, the study by Brændvik et al. (2016) found that 8 weeks strength training was less effective than treadmill training in improving FAP (*p* = 0.844) in pwMS. Within-group analyses showed significant or nearly significant improvements (*p* = 0.025 for the TT group and *p* = 0.061 for the ST group) [16].

### Aerobic and cardiovascular training

Aerobic and cardiovascular training were also widely investigated, primarily for their impact on cardiorespiratory endurance, fatigue resistance, and overall walking performance. Caravaca et al. (2022) reported improvements in both gait speed (16.9%) and walking endurance (25.5%) after 10 weeks of high-intensity concentric resistance training, emphasizing the importance of rapid, forceful muscle contractions that more closely mimic real-world walking demands [26].

Similarly, in the study by Galperin et al. (2023), after 6 weeks of treadmill training, participants in the treadmill training with virtual reality group (TT + VR) and treadmill training alone group (TT) showed changes in walking outcomes. The T25FW decreased by 2.9% in TT (*p* = 0.176) and 7.7% in TT + VR (*p* < 0.001), while the 6-minute walk distance increased in both groups (TT: +20.7 m, *p* = 0.021; TT + VR: +14.4 m, *p* = 0.093). Usual walking speed improved in both groups (*p* < 0.001) and persisted at 3 months. Cadence and dual-task step regularity also increased (*p* < 0.001), with dual-task regularity improving only in TT and cadence in TT + VR, although these gains did not persist at follow-up [24].

In the study by Peruzzi et al.(2017), both TT and VR + TT groups showed significant improvements after 6-week intervention in spatiotemporal gait parameters, with no significant between-group differences. Lower limb joint ROM increased in both groups, with the VR + TT group showing significant gains in knee and hip ROM on the more-affected side (knee ST: *p* = 0.013; hip ST: *p* = 0.001; hip DT: *p* = 0.001).Joint-generated powers at the hip and ankle improved in both groups, with greater hip flexor power gains on the MA side in the VR-TT group (ST: *p* = 0.004; DT: *p* = 0.007) [4].

In contrast, Pau et al. (2018) found that a 6-month adapted physical activity program led to a 23.4% increase in gait speed, with moderate improvements in stride length and cadence, although overall gait kinematics remained largely unchanged [25].

### Balance and sensory motor training

Balance-focused protocols aimed at improving postural control and reducing fall risk also showed promising results. Kalron et al. (2019) found that 12 weeks of Pilates improved step length(*P* = 0.023), single support phase *(P* = 0.008), and walking speed (*p* = 0.018), although these gains were comparable to those achieved through conventional physical therapy [20].

Sepehrifar et al. (2023) further emphasized this point, demonstrating that multi-functional swing suspension training after 8-week training produced greater gains in walking speed for participants with higher disability levels (EDSS 4.5–6.5) [2]. In the study by Nilsagård et al. (2013), participants completed a 7-week balance exercise program, results showed that in the exercise group, MSWS-12 improved significantly (*p* ≤ 0.01), with large effect sizes [21].

In the study by Escudero et al. (2017), the WBV (whole body vibration) group showed significant improvements in most gait parameters, including FAP(Functional Ambulation Profile), velocity, step length, step time, stance time, double support time, and step length asymmetry (*p* < 0.05). Clinically, WBV outperformed control group in FAP (p = 0.005), velocity (*p* = 0.003), step length (*p* = 0.007), and step length asymmetry (*p* = 0.028), with no differences compared to balance training group and no changes in base of support [22].

### Cognitive-motor and dual-task training

Cognitive-motor training, particularly dual-task approaches, has gained attention as a means of integrating cognitive and motor functions, critical for real-world ambulation.

Tramontano et al. (2023) compared conventional neuromotor training with a dual-task paradigm involving simultaneous cognitive and motor exercises. The dual-task approach improved gait smoothness and dynamic postural stability, with IMU-based assessment showing significant between-group differences favoring the DTg (dual-task cognitive-motor training) group. During the 10mWT, the DTg group demonstrated greater stability and smoothness at T1 (post-intervention), while the CTg (conventional therapy) group showed no significant changes [27].

Galperin et al. (2023) reported that dual-task step regularity improved in the TT group (*p* = 0.011), while cadence improved in the TT + VR group (*p* = 0.001), but these effects were not sustained at 3-month follow-up [24].

### Combined training protocols

Motl et al. (2012) reported that 8 weeks of combined training produced notable gait improvements, including increases in FAP score (+ 7%, ES = 0.64), gait velocity (+ 10%, ES = 0.71), and stride length (+ 7%, ES = 0.85). Smaller gains were observed for single support (+ 3%, ES = 0.55), swing phase (+ 3%, ES = 0.55), and double support (+ 5%, ES = 0.48), while other gait parameters remained unchanged [17].

Similarly, Gutiérrez-Cruz et al. (2020) found that combined training for 24 weeks reduced double-support time by 7.1% and increased single-support time by 2.1%, reflecting improved dynamic stability [1].

Davies et al. (2016) reported that participants completed training twice a day, five days per week, for six weeks. Both the MAC (Motor Adaptation Cohort), which consisted of balance and gait training, and the TEC (Therapeutic Exercise Cohort), similar to a traditional exercise program, showed significant improvements in walking endurance (6MWT: *p* = 0.002), walking velocity (*p* = 0.004), and step length (*p* < 0.001) after the interventions. The average percent changes were 14.5% for endurance, 14.6% for velocity, and 11.4% for step length, with no significant differences between groups or interactions (*p*  > 0.01). Step width and cadence were unaffected (*p*  > 0.01) [23].

**Table 2:** Summary of exercise interventions and key outcomes in people with multiple sclerosis

Author (Year)	Study Design	Population (M/F)	EDSS/PDDS	Age (Mean ± SD)	MS Subtype	Intervention 1 (Duration/Frequency)	Intervention 2 (Duration/Frequency)	Control Group	Instrumentation	Key Outcomes	Effect
Conroy (2018)[19]	RCT	9:18	PDDS 2–6	52.7 ± 13.2	RR, SP, PP	Tele-managed home exercise (6 months, 3x/week, 30 min)	Routine home exercise (6 months, 3x/week, 30 min)		None	↑ 6MWT, MSWS-12	Positive
Nilsagård (2013)[21]	RCT	32:10	Not reported	50.0 ± 11.5	RR, SP, PP	Wii Fit Plus^®^ video game (6–7 weeks, 12 sessions, 30 min)	None	No exercise	None	↑ MSWS-12, DGI, T25FW	Positive
Tramontano (2023)[27]	RCT	0:39	EDSS 0–6.5	CTg: 51.6 ± 9.4, DTg: 50.4 ± 10.3	RR, SP	Conventional neuromotor rehab (4 weeks, 3x/week, 50 min)	Dual-task motor-cognitive therapy (4 weeks, 3x/week, 50 min)		IMUs	↑ WS, AD step, Freq stride, AD stride	Positive
Sepehrifar (2023)[2]	RCT	0:47	EDSS 2–6.5	36.87 ± 6.31 36.55 ± 3.74	RR	Mobility, strength, flexibility training (8 weeks, 3x/week, 45 min)	None	Attentional control	None	↑T25FW (More in moderate disability)	Positive
Gutierrez (2005)[13]	Non-RCT	7:1	EDSS 2.5–5.5	46.0 ± 11.5	RR	Resistance training (8 weeks, 2x/week, 60 min)	None		Force platform	↓Knee ROM, ↑stride time, stance time, step length, velocity	Positive
Galperin (2023)[24]	RCT	7:3	EDSS 2–6	TT: 49.1 ± 9.7, TT + VR: 49.0 ± 10.0	RR	Treadmill training (6 weeks, 3x/week, 30–45 min)	Treadmill + virtual reality (6 weeks, 3x/week, 30–45 min)		wearing sensors accelerometer	dual-tasking step (↑ in the TT group) speed, UW speed, dual-tasking cadence (↑ in the TT + VR) group regularity, T25FW, 6MWT	Positive
Alashram (2024)[8]	RCT	9:15	EDSS ≤ 5.5	Telko + CPT: 37.8 ± 10.8, CPT: 35.2 ± 9.3	PP, SP, PR	CPT + gait training (4 weeks, 3x/week, 20 min)	CPT + Telko device (4 weeks, 3x/week, 35 min)		None	↑6MWT, WS	Positive
Filipi (2010)[14]	Non-RCT	11:22	EDSS 1–6.5	38.8 ± 10.7	RR, SP, PP	Resistance exercise, EDSS 1–4 (6 months, 2x/week, 50 min)	Resistance exercise, EDSS 4.5–6.5 (6 months, 2x/week, 50 min)		3D gait-analysis, force platform	↑Joint kinematics, ground reaction forces, joint angles	Positive
Brændvik (2016)[16]	RCT	4:7	EDSS 1–6	TG: 46.6 ± 6.2, SG: 49.1 ± 7.4	RR, PP, SP	Treadmill group (8 weeks, 3x/week, 30 min)	Strength group (8 weeks, 3x/week, 30 min)		GAITRite, Accelerometer	↑FAP, WWE, APaccRS, MLaccRS (in TT group), (VaccRMS↓ in TT),	Positive
Motl (2012)[17]	Non-RCT	5:8	EDSS 4–6	51.5 ± 11.3	RR, SP, PP	Aerobic + resistance + balance (8 weeks, 3x/week, 60 min)	None		GAITRite,	↑MSWS-12, T25FW, FAP, gait ↔velocity, cadence, stride length DST	Positive
Escudero (2017)[22]	Non-RCT	WBV: 6:10, BT: 5:9	EDSS 0–4.5	WBV: 43.1 ± 10.2, BT: 40.3 ± 8.9	RR	Whole-body vibration + aerobic/circuit (12 weeks, 2x/week, 60 min)	Balance trainer + aerobic/circuit (12 weeks, 2x/week, 60 min)	No exercise	GAITRite,	↑FAP, velocity, step length, base of support	Positive
Heine (2019)[18]	Non-RCT	4:6	EDSS 2.5–4	48.8 ± 8.9	PP, SP, RR	PRT + treadmill (16 weeks, 3x/week, 30–60 min)	None	No exercise	None	↑Total distance, WS, step length, ↔stance phase, ECWgross	Positive
Kalron (2019)[20]	RCT	PT: 8:14, Pilates: 8:15	EDSS 3–6	PT: 44.3 ± 6.6, Pilates: 42.9 ± 7.2	RR	Pilates + home exercise (12 weeks, 1x/week + daily 15 min)	Physiotherapy + home exercise (12 weeks, 1x/week + daily 15 min)		Instrumented treadmill	↑ 6MWT, velocity, cadence, step time, stride length	Positive
Gutiérrez-Cruz (2020)[1]	RCT	8:9	EDSS 0–6	EX: 40.7 ± 8.2, CG: 47.2 ± 9.8	RR	Combined training (24 weeks, 3x/week, 60 min)	None	Normal activity	Instrumented walk way, Force platform	↑ double-support time, step length, ↔ DTC	Positive
Callesen (2020)[15]	RCT	2:4.1	EDSS 2–6	> 18	RR, PP, SP	Progressive resistance training (10 weeks, 2x/week, 60 min)	Balance/motor control training (10 weeks, 2x/week, 60 min)	Usual care	None	1.BMCT vs. CG: - T25FW, SSST ↑ 6minWT, ↔ 2. PRT vs. CG: - T25FW, SSST, 6minWT, ↔	BMCT = Positive PRT = ineffective
Caravaca (2022)[26]	RCT	15:15	EDSS 1–6	46.2 ± 10.4	RR, SP	Lower-limb FVCRT (10 weeks, 3x/week, 45 min)	None		None	↑10MWT, 6MWT	Positive
Peruzzi (2017)[4]	RCT	TT: 7:4, TT + VR: 6:8	EDSS 3–5.5	TT: 42.0 ± 12.0, TT + VR: 43.6 ± 10.2	RR	VR + treadmill training (6 weeks, 3x/week, 30 min)	Treadmill training (6 weeks, 3x/week, 30 min)		inertial sensors, force plat, Motion Capture, Force platform	↑6MWT in both groups (without a between-group difference), ↑10MWT in the VR-TT group (without between-group differences). ↑gait velocity, cadence, stride length under single- and dual-task conditions (without between group differences) length, gait speed	Positive
Pau (2018)[25]	RCT	6:5	EDSS 2.5–4.5	APA: 47.4 ± 10.8, CG: 44.5 ± 13.5	RR	APA training (24 weeks, 3x/week, 60 min)	None	No exercise	Motion Analysis System, Force platform	↑Stride length, velocity, and cadence ↔stance phase, swing phase	Spatiotemporal = Positive Kinematic = ineffective
Dodd (2011)[29]	RCT	10:26	Not reported	PRT: 47.7 ± 10.8, CG: 50.4 ± 9.6	RR	Progressive resistance training (10 weeks, 2x/week, 60 min)	None	Social program	None	↑Fast WS, 2MWT	Positive
Manca(2020)[28]	RCT	10:2 9:4	EDSS 2-5.5	CST 42.8 ± 15.3 DST 49.2 ± 9.1	RR	CST(six weeks, 3X /week, 25 min)	DST(six weeks, 3X /week, 25 min)		Motion Analysis System, Force platform	DST: ↑ velocity, stride length, stride time, cadence, and the peak of ankle-generated power ↔ Ankle moment and ROM CST: ↔Velocity, stride length, stride time, cadence, the peak of ankle generated power, ankle moment, and ROM	Positive
Davies(2016)[23]	Not RCT	7:8 12:5	EDSS 3.0-6.5	MAC 52.6 ± 9 TEC 54.0 ± 9	RR SR	MAC)twice a day ,60 min on five consecutive days each week (	TEC(twice a day ,60 min on five consecutive days each week)		GAITRite	↑ velocity, step width, step length, Cadence (between groups↔)	Positive

Legend

•Study Design: RCT = Randomized Controlled Trial, Non-RCT = Non-Randomized Controlled Trial

•EDSS/PDDS: Expanded Disability Status Scale/Patient Determined Disease Steps

•MS Subtype: RR = Relapsing-Remitting, SP = Secondary Progressive, PP = Primary Progressive, PR = Progressive Relapsing

•Interventions: PRT = Progressive Resistance Training, CPT = Cognitive-Physical Training, FVCRT = Fast-Velocity Concentric Resistance Training, APA = Adapted Physical Activity, CST = Contralateral strength training, DST = direct strength taining, MAC = Motor adaptation cohort, TEC = Therapeutic exercise cohort,

• Outcomes

•6MWT = 6-Minute Walk Test, T25FW = Timed 25-Foot Walk, MSWS-12 = Multiple Sclerosis Walking Scale-12

•DGI = Dynamic Gait Index, WS = Walking Speed, AD = Average Duration, Freq = Frequency

•DTW/UW = Dual-Task Walking/Usual Walking, FAP = Functional Ambulation Profile, WWE = Walking Work Economy

•APaccRMS/VaccRMS/MLaccRMS = Acceleration Root Mean Square (Anteroposterior/Vertical/Mediolateral)

•ECWgross = Energy Cost of Walking, GCT = Gait Cycle Time, DTC = Dual-Task Cost, SSST = Six Spot Step Test

•10MWT = 10-Meter Walk Test, 2MWT = 2-Minute Walk Test. DST = Double support time

Effect: Positive = Statistically significant improvement in at least one gait parameter

Notes

•Intervention durations and frequencies were standardized for clarity (e.g., “3x/week” instead of “3 days per week”)

•Outcomes were grouped to reflect functional (e.g., 6MWT), biomechanical (e.g., stride length), and patient-reported (e.g., MSWS-12) measures

•The “Effect” column summarizes the overall impact based on reported results, assuming positive effects where improvements were note

## Discussion

The findings from this narrative review underscore the critical role of structured exercise interventions in improving gait performance in people with multiple sclerosis (pwMS). Gait dysfunction, characterized by reduced walking speed, shorter stride length, impaired joint range of motion, and compromised balance, is a significant contributor to disability and reduced quality of life in this population. Effective rehabilitation strategies are therefore essential for mitigating mobility impairments and enhancing functional independence [1, 4].

One of the most consistent observations across the reviewed studies was the impact of different types of resistance exercise training on spatiotemporal and kinematic gait parameters. Resistance training was shown to improve joint torque, ground reaction forces, and muscle strength, leading to enhanced gait stability and propulsion. For instance, Filipi et al. (2010) reported findings consistent with these results, indicating that resistance training in pwMS with mild to moderate disability led to improvements in gait dynamics and postural control [14].

Although interventions focused on aerobic and cardiovascular training primarily targeted cardiorespiratory endurance and fatigue resistance. Studies like Caravaca et al. (2022) highlighted the benefits of high-velocity concentric resistance training, which produced substantial improvements in both gait speed (16.9%) and walking endurance (25.5%) in patients with mild to moderate disability. These findings are consistent with the principles of muscle power training, which prioritize rapid, forceful contractions that more closely mimic the demands of real-world walking [26]. However, such gains often require sustained engagement, as demonstrated by Pau et al. (2018), where a 6-month adapted physical activity program led to a 23.4% increase in gait speed. These findings underscore the importance of long-term, high-intensity training for achieving sustained functional gains, particularly in individuals with moderate to severe disability [25].

Balance and sensory motor training also emerged as critical components of gait rehabilitation, particularly for individuals with moderate to severe disability. Kalron et al. (2019) reported that Pilates-based training improved step length, single support phase, and walking speed, although these gains were comparable to those achieved through conventional physical therapy. This suggests that while Pilates can enhance core stability and neuromuscular coordination, more intensive balance-specific protocols may be required for substantial gait improvements [20]. Sepehrifar et al. (2023) further emphasized this point, demonstrating that multi-functional swing suspension training produced greater gains in walking speed for participants with higher disability levels (EDSS 4.5–6.5). This aligns with the concept that more challenging, task-specific balance exercises are necessary to induce meaningful neural adaptations in this population [2]. In the study by Nilsagård et al. (2013), a 6–7-week Nintendo Wii Fit^®^ program for people with MS showed high adherence and good acceptability. The exercise group demonstrated reduced perceived walking limitations (MSWS-12) and improved TUG-cognitive, while TUG and Timed 25-Foot Walk remained unchanged. The findings support the positive role of Wii-based exercises in balance improvement and highlight the importance of exercise intensity for stronger effects [21].

The integration of cognitive and motor training represents another promising approach for addressing the complex, multifactorial nature of gait impairment in MS. Dual-task training, as investigated by Tramontano et al. (2023) investigated combined motor and cognitive therapy (DTg) versus dynamic postural stability rehabilitation (CTg) in pwMS with an 8-week follow-up. DTg showed greater improvements in dynamic balance (Mini-BESTest), postural responses, and gait stability, smoothness, and symmetry. During straight and curved walking, DTg outperformed CTg in spatiotemporal and smoothness metrics (LDLJv, nRMS). In blindfolded walking, both groups improved, but DTg also reduced visual dependence. This IMU-based study highlights the benefits of integrated cognitive-motor training for gait and balance recovery in pwMS [27]. also Galperin et al. (2023), offers the potential to simultaneously enhance cognitive processing and motor coordination, critical for navigating complex, real-world environments. These studies found improvements in gait smoothness, step regularity, and dynamic postural stability, although some gains were not sustained at follow-up, highlighting the need for continuous cognitive-motor engagement. This is particularly relevant given the executive function deficits commonly observed in pwMS, which can significantly impair dual-task performance [24].

Combined training protocols, integrating resistance, aerobic, balance, and cognitive elements, generally produced the most comprehensive benefits. For example, Motl et al. (2012) demonstrated that combined training improved walking mobility (T25FW) and spatiotemporal parameters, including stride length and single support phase, with moderate to large effect sizes. This approach aligns with the concept of multimodal training, which targets multiple physiological systems and neural pathways, potentially offering superior outcomes compared to single-modality interventions [17]. Davies et al. (2016), showed that both the MAC (Motor Adaptation Cohort) and TEC (Therapeutic Exercise Cohort) protocols led to significant improvements in postural balance and mobility in people with MS, with no significant differences between the groups. These findings highlight the importance of high-frequency physical therapy in promoting balance and mobility gains, even when exercises are less focused or performed at home. Increases in step length and walking speed in both groups suggest that consistent and frequent walking activities can positively influence mobility.

These findings align with Kalron et al.(2015), who observed that a 3-week, high-frequency, multi-modal rehabilitation program led to improvements in mobility for people with MS, even though most gains were modest and fell below the minimal clinically important threshold. This suggests that both the frequency and intensity of exercise are crucial factors for optimizing motor function in pwMS and should be carefully considered when designing rehabilitation programs [30].

The improvements in gait biomechanics observed in PwMS following structured exercise interventions are driven by neuromechanical adaptations, encompassing neuroplasticity, enhanced motor unit recruitment, and improved proprioceptive control. Neuroplasticity, the brain’s ability to reorganize neural pathways, is a key mechanism facilitating functional recovery. Aerobic and resistance training upregulate brain-derived neurotrophic factor (BDNF), promoting synaptic remodeling and cortical reorganization in motor and sensory cortices, which enhance gait coordination and reduce variability [6, 7]. For example, Uzunoğlu et al. (2025) demonstrated that vestibular rehabilitation fosters neuroplastic adaptations, improving kinematic parameters such as joint angles and range of motion [6]. Resistance training enhances motor unit recruitment by increasing the activation of high-threshold motor units, resulting in greater joint torque and stability during walking, as evidenced by Filipi et al. (2010) [13]. Proprioceptive control, essential for dynamic balance and gait smoothness, is improved through balance and sensorimotor training, which strengthen sensory feedback loops and cerebellar integration, as shown by Kalron et al. (2017) [19]. Cognitive-motor dual-task training further supports executive function by enhancing prefrontal cortex activity, critical for motor planning and dual-task performance during ambulation, as reported by Morelli et al. (2021) and Kalron et al. (2017) [10, 19]. These neuromechanical mechanisms collectively contribute to the observed improvements in spatiotemporal, kinematic, and kinetic gait parameters, emphasizing the value of multimodal, task-specific exercise protocols in promoting lasting neuromuscular adaptations in PwMS.

### Limitations

Despite these promising findings, several limitations should be considered when interpreting the results of this review.

First, there was significant heterogeneity in study designs, intervention protocols, and outcome measures, complicating direct comparisons and meta-analytic synthesis. The included studies varied widely in terms of training intensity, duration, and frequency, as well as the types of gait parameters assessed. This lack of standardization limits the ability to draw definitive conclusions about optimal training protocols.

Second, many studies lacked long-term follow-up, making it difficult to assess the sustainability of observed improvements. Given the progressive nature of MS, continuous training may be necessary to maintain functional gains over time. Future studies should incorporate longer follow-up periods to better evaluate the durability of these interventions.

Third, sample sizes in many studies were relatively small, reducing statistical power and generalizability. This is particularly problematic given the heterogeneity of the MS population, which includes varying disease subtypes (RRMS, SPMS, PPMS) and disability levels. Larger, multicenter trials are needed to confirm the efficacy of these interventions across diverse patient populations. Fourth, this work is a narrative review, it did not follow a fully systematic search strategy. Consequently, there is a possibility that some relevant studies may have been unintentionally omitted.

Due to the insufficient and inconsistent information regarding patient characteristics and their relationship with interventions and outcomes, the ability to perform a precise analysis of the interactions between intervention types and patient profiles was limited. This issue represents a significant limitation in comprehensively evaluating the effects of the interventions and highlights the need for future studies with more complete and systematic data.

Additionally, few studies comprehensively assessed cognitive function despite its known impact on gait control. Given the central role of executive function in coordinating complex motor tasks, future studies should prioritize the inclusion of cognitive assessments and consider integrating neuropsychological training as part of gait rehabilitation protocols.

Finally, many studies did not control for potential confounding variables, such as medication use, disease duration, and baseline physical activity levels, which can influence gait outcomes. More rigorous trial designs, including randomized controlled trials with blinded assessors, are needed to address these methodological limitations.

## Conclusion

This review highlights the substantial potential of structured exercise interventions to improve gait function in pwMS. High-intensity, task-specific training, combined with cognitive and balance exercises, appears to offer the most comprehensive benefits. However, the heterogeneity of study designs, small sample sizes, and lack of long-term follow-up underscore the need for more standardized, large-scale RCTs. Future research should prioritize personalized, progressive training approaches, incorporating advanced technologies, such as wearable sensors and AI-driven gait analysis, to optimize rehabilitation outcomes and improve functional independence in this population.

## Supplementary Information

Below is the link to the electronic supplementary material.

Supplementary Material 1

Supplementary Material 2

Supplementary Material 3

## Data Availability

No datasets were generated or analysed during the current study.

## Abbreviations

MS: Multiple Sclerosis
RRMS: Relapsing-Remitting Multiple Sclerosis
SPMS: Secondary Progressive Multiple Sclerosis
PPMS: Primary Progressive Multiple Sclerosis
EDSS: Expanded Disability Status Scale
PDDS: Patient Determined Disease Steps
PRISMA: Preferred Reporting Items for Systematic Reviews and Meta-Analyses
PICOS: Population, Intervention, Comparator, Outcomes, Study design
ROM: Range of Motion
MSWS: Multiple Sclerosis Walking Scale
VR: Virtual Reality
ST: Single task
DT: Dual task
CPT: Conventional physical therapy
WBV: Whole Body Vibration
