# Supplementary material for: Biomechanical insights into gait rehabilitation for multiple sclerosis: a narrative review of exercise modalities and progressive training approaches
Source: BMC Sports Sci Med Rehabil. 2025 Oct 14;17:297. doi: 10.1186/s13102-025-01339-4 (PMC12522390; doi:10.1186/s13102-025-01339-4)
Supplement: Supplementary file 1 — Supplementary Material 1 [file 13102_2025_1339_MOESM1_ESM.pdf]

- Supplementary File 1. Tables presenting detailed results of quality assessment (CASP checklist)

	Study Type	Quality Score (CASP)	Blinding	Key Findings
Carmen Gutiérrez-Cruz	RCT	9/11	None	Stratified randomization, significant results, 24-week follow-up.
Irina Galperin	RCT	10/11	Single-blind	Randomization, significant results, 3-month follow-up.
Jacob Callesen	RCT	10/11	Single-blind	Cluster randomization, reliable results, multi-center design.
Luis Andreu-Caravaca	RCT	10/11	Single-blind	Randomization, significant results, single-blind.
Ylva E Nilsagård	RCT	9/11	Single-blind	Randomization, positive results, single-blind.
Susan S Conroy	RCT	9/11	Single-blind	Randomization, no significant differences, single-blind.
Agnese Peruzzi	RCT	9/11	Single-blind	Randomization, significant results, innovative design.
KJ Dodd	RCT	10/11	Single-blind	Randomization, short-term positive results, 22-week follow-up.
Massimiliano Pau	RCT	8/11	None	Randomization, improved spatio-temporal parameters, no blinding.
Marco Tramontano	RCT	10/11	Single-blind	Randomization, significant results (stability, smoothness), 8-week follow-up.
Alon Kalron	RCT	10/11	Single-blind	Randomization, improved walking speed, single-blind.
Anas R. Alashram	RCT	9/11	Single-blind	Randomization, improved balance and functional capacity, single-blind.
Sara Sepehrifar	RCT	10/11	Single-blind	Randomization, significant improvements in limb function and quality of life, single-blind.
Siri Merete Brændvik	RCT	10/11	Single-blind	Randomization, significant improvement in treadmill group, single-blind.
Shahid Escudero-Uribe	RCT	10/11	Single-blind	Randomization, significant improvement in gait parameters (WBV), single-blind.
Andrea Manca	RCT	9/11	Unclear	Randomization, significant strength gains in both groups, improved gait speed in DST, unclear blinding.
