# Supplementary material for: Biomechanical insights into gait rehabilitation for multiple sclerosis: a narrative review of exercise modalities and progressive training approaches
Source: BMC Sports Sci Med Rehabil. 2025 Oct 14;17:297. doi: 10.1186/s13102-025-01339-4 (PMC12522390; doi:10.1186/s13102-025-01339-4)
Supplement: Supplementary file 2 — Supplementary Material 2 [file 13102_2025_1339_MOESM2_ESM.pdf]

- Supplementary File 2. Tables presenting detailed results of quality assessment (MINORS checklist)

First Author	Study Type	Quality Score (MINORS)	Blinding	Key Findings
Gregory M. Gutierrez	Prospective Cohort	10/16	None	Improved gait kinematics and strength, no control group.
Robert W. Motl	Prospective Pilot	10/16	None	Improved walking mobility, no control group.
Mary L. Filipi	Prospective Cohort	11/16	None	Improved balance and gait, no control group, 27% dropout.
Brenda L. Davies	Prospective Cohort	10/16	None	Improved balance and mobility, no control group.
Martin Heine	Prospective Pilot	10/16	None	Improved walking distance, increased ankle push-off power in less-affected leg, no control group.
